# Fitness to Drive Policy in Inpatient Setting: Findings of QI Project

**DOI:** 10.1192/bjo.2024.392

**Published:** 2024-08-01

**Authors:** Sajid Mahmood, Heba Radwan, Omotilewa Omotoso, Waqqas Khokhar, Nosheen Shams

**Affiliations:** Leicestershire Partnership NHS Trust, Leicester, United Kingdom

## Abstract

**Aims:**

Background: Nature and degree of mental illness can impair abilities of patients to drive safely which puts their own safety and safety of others at risk. There is also an ongoing concern of patients not being properly informed on their duty to inform the DVLA and potentially to stop driving for an extended period.

Aims:

1. To assess if risk assessment of patients in term of driving status was completed at time of admission, during their stay on ward, and if any advice regarding fitness to drive was given at time of discharge.
2. To improve patients being asked about driving status on admission to 100% of patients.
3. To improve rates of service users being informed of the DVLA guidance following a mental health illness to 100% of patients.

**Methods:**

It is a Quality Improvement (QI) project. Baseline information on current practices were assessed against local fitness to drive policy of Leicestershire Partnership NHS Trust in May 2023. Data was collected from 10 inpatient (6 general adult & 4 old age) wards. All patients who were discharged in the month of January 2023 were included for audit. Information was collected about driving status of patients at time of admission, during their stay on ward, and if any advice regarding fitness to drive was given at time of discharge. Data was recorded anonymously. Results are reported in percentages for descriptive statistics.

**Results:**

Risk assessment was completed in 95% of patients on admission. About 12% (15/128) of the patients were driving at the time of admission, 80% of them were female. Assessment of driving risk during admission only took place in 11.7% (13/128) of cases. Advice on fitness to drive at time of discharge was given only in 12.5% (16/128) of cases. About ¼ of patients who were driving at time of admission, did not receive advice on fitness to drive at time of discharge from hospital.

**Conclusion:**

There is a huge gap in clinical practice regarding compliance with fitness to drive policy. There is an urgent need to improve awareness among mental health teams that they have a role with regard to assessment of their patients’ risks and fitness to drive. An educational training video will be prepared and shared with clinicians in December 2023 to fill gaps in the service. Further information will be collected on practices related to fitness to drive policy in March 2024 for further evaluation of services.

